# A Chimeric NaV1.8 Channel Expression System Based on HEK293T Cell Line

**DOI:** 10.3389/fphar.2018.00337

**Published:** 2018-04-06

**Authors:** Xi Zhou, Yunxiao Zhang, Dongfang Tang, Songping Liang, Ping Chen, Cheng Tang, Zhonghua Liu

**Affiliations:** The National and Local Joint Engineering Laboratory of Animal Peptide Drug Development, College of Life Sciences, Hunan Normal University, Changsha, China

**Keywords:** NaV1.8, functional expression, HEK293T, pharmacology, APETx-2, MrVIB

## Abstract

Among the nine voltage-gated sodium channel (NaV) subtypes, NaV1.8 is an attractive therapeutic target for pain. The heterologous expression of recombinant NaV1.8 currents is of particular importance for its electrophysiological and pharmacological studies. However, NaV1.8 expresses no or low-level functional currents when transiently transfected into non-neuronal cell lines. The present study aims to explore the molecular determinants limiting its functional expression and accordingly establish a functional NaV1.8 expression system. We conducted screening analysis of the NaV1.8 intracellular loops by constructing NaV chimeric channels and confirmed that the NaV1.8 C-terminus was the only limiting factor. Replacing this sequence with that of NaV1.4, NaV1.5, or NaV1.7 constructed functional channels (NaV1.8/1.4L5, NaV1.8/1.5L5, and NaV1.8/1.7L5, respectively), which expressed high-level NaV1.8-like currents in HEK293T cells. The chimeric channel NaV1.8/1.7L5 displayed much faster inactivation of its macroscopic currents than NaV1.8/1.4L5 and NaV1.8/1.5L5, and it was the most similar to wild-type NaV1.8 expressed in ND7/23 cells. Its currents were very stable during repetitive depolarizations, while its repriming kinetic was different from wild-type NaV1.8. Most importantly, NaV1.8/1.7L5 pharmacologically resembled wild-type NaV1.8 as revealed by testing their susceptibility to two NaV1.8 selective antagonists, APETx-2 and MrVIB. NaV chimeras study showed that at least the domain 2 and domain 4 of NaV1.8 were involved in binding with APETx-2. Our study provided new insights into the function of NaV1.8 intracellular loops, as well as a reliable and convenient expression system which could be useful in NaV1.8 studies.

## Introduction

Mammalian NaV is composed of four homologous domains, each domain contains six transmembrane segments, in which the first four segments (S1 – 4) construct the VSM and the last two (S5 – 6) constitute the PM (Catterall, 2012, 2014b). PMs from all four domains of the channel construct the central ion conducting pathway, which is surrounded by VSMs. When the membrane is depolarized, the S4 segment, which carries the gating charges, moves outward to allosterically open the central pore. Recent landmark progresses regarding the high resolution crystal structures of bacterial sodium channels and cryo-EM structures of cockroach Na_V_PaS and eel NaV1.4 have provided important insights to understand the structure-function relationship of mammalian NaVs (Payandeh et al., 2011, 2012; Zhang et al., 2012; Shen et al., 2017; Yan et al., 2017). In mammals, nine NaV subtypes have been characterized so far, and their roles in pathological conditions such as epilepsy, pain, ataxia and so on, have been verified by numerous studies (Catterall, 2014a; Emery et al., 2016; Schwarz et al., 2016). Among them, NaV1.8 channel is a major contributor to action potential (AP) generation and propagation in peripheral primary sensory neurons, and it was deemed as an ideal target for pain-treating drugs (Blair and Bean, 2003). The pivotal role of NaV1.8 in the pain transduction pathway was supported by gene knocking out studies, in which knocking out the NaV1.8 gene or genes of several NaV1.8 regulatory proteins in mouse attenuated the pain behavior (Akopian et al., 1999; Foulkes et al., 2006; Zhang and Verkman, 2010). Actually, several NaV1.8 selective antagonists characterized in recent years showed analgesic effect in animal pain models (Jarvis et al., 2007; Bagal et al., 2015; Payne et al., 2015).

The functional expression of NaV1.8 channel in mammalian cell lines is an important approach to study its electrophysiological and pharmacological properties. For this goal, the cell lines ND7/23 and SH-SY5Y were commonly used for functional NaV1.8 currents expression by using the transient transfection method (John et al., 2004; Dekker et al., 2005). And another choice is *Xenopus* oocytes, but it needs *in vitro* transcription and microinjection of NaV1.8 mRNA (Rabert et al., 1998). In recent years, commercial cell lines stably expressing NaV1.8 channel were available and were amenable to the drug high-throughput screening (HTS) method, such as the recombinant HEK293T cell line co-expressing the human NaV1.8 and β1 accessory subunit (Merck Millipore, Billerica, MA, United States). In this cell line, the mean NaV1.8 current was -900 ± 17 pA. Compared with the successful heterologous expression of functional NaV1.3 – 1.5 and NaV1.7 currents in non-neuronal cell lines such as CHO-K1, COS-7 and HEK293T, transient transfection of NaV1.8 channel into them yielded no or low-level functional currents, even though it was co-expressed with the β subunits (John et al., 2004; Zhang et al., 2008). We speculated that some parts of the NaV1.8 protein sequence might be responsible for this. The ND7/23 cell line supported the functional expression of NaV1.8 channel, possibly because it has a background of dorsal root ganglion neuron and contains accessory proteins interacting with these sequences.

Actually, some signals in the NaV1.8 protein sequence preventing its surface trafficking were identified in previous studies (Zhang et al., 2008; Li et al., 2010). And proteins such as β3 subunit, annexin II light chain p11, PDZD2, contactin and calnexin were shown to regulate the functional expression of NaV1.8 (Okuse et al., 2002; Rush et al., 2005; Zhang et al., 2008; Shao et al., 2009; Li et al., 2010). Nonetheless, destroying or masking the identified signals, or co-expressing several of these partner proteins with NaV1.8 channel had not achieved high-level functional currents expression in non-neuronal cell lines. Choi et al. (2004) and Vijayaragavan et al. (2004) had locked an unidentified retention/retrieval signal in the C-terminus of NaV1.8 by conducting chimeric channel studies. In ND7/23 cells, the chimeric channel by replacing NaV1.8 C-terminus with that of NaV1.4 (NaV1.8/1.4C) showed approximately 2-folds increase of the current density, while the reverse chimera NaV1.4/1.8C showed approximate 7-folds decrease of its current when compared with the parental NaV1.4 channel (Choi et al., 2004). And replacing the NaV1.8 C-terminus with that of NaV1.7 constructed chimeric channel functionally expressed in tSA201 cells, the peak current was approximately 1 nA (Vijayaragavan et al., 2004). These studies suggested that the NaV1.8 C-terminus is an important factor regulating its functional expression. However, the roles of the other four intracellular loops in NaV1.8 were not systematically analyzed yet.

The present study showed that replacing NaV1.8 C-terminus with that of NaV1.4, NaV1.5, or NaV1.7 constructed functional channels (NaV1.8/1.4L5, NaV1.8/1.5L5, and NaV1.8/1.7L5, respectively), which expressed high-level NaV1.8-like currents in HEK293T cells. By conducting a screening analysis of the NaV1.8 intracellular loops, we confirmed that the C-terminus was the only limiting factor. Most importantly, the chimeric channel NaV1.8/1.7L5 pharmacologically resembled wild-type NaV1.8 as revealed by testing their susceptibility to two NaV1.8 selective antagonists, MrVIB and APETx-2 (Ekberg et al., 2006; Blanchard et al., 2012; Peigneur et al., 2012). The present study provided new insights into the function of NaV1.8 intracellular loops and this chimeric NaV1.8 expression system could be useful in the electrophysiological and pharmacological studies.

## Materials and Methods

### Toxins and Hazard Waste Treatment

MrVIB was a kindly gift from professor Paul Alewood (Institute for Molecular Bioscience, The University of Queensland, Australia). APETx-2 was purchased from Abcam (Abcam PLC, Cambridge, United Kingdom). These neurotoxins were used in laboratory only and the hazardous wastes were collected and sent for centralized treatment by Hunan Normal University.

### Primary Culture of Rat Dorsal Root Ganglion (DRG) Neurons

SD rats (Hunan SJA Laboratory Animal Co., Ltd., Changsha, Hunan, China) were used according to the guidelines of the National Institutes of Health for care and use of laboratory animals. The experiments were approved by the Animal Care and Use Committee of the College of Medicine, Hunan Normal University. Acutely dissociated DRG neurons were prepared from 4-weeks old SD rats and maintained in short-term primary culture by using the method described by Hu and Li (1996).

### Molecular Cloning

NaVs in this study were kindly gifts from Dr. Theodore R. Cummins (Stark Neurosciences Research Institute, Indiana University School of Medicine, United States). hNaV1.5 and rNaV1.8 cDNAs were subcloned into a pCMV-Blank vector (Beyotime, Shanghai, China) between sites Hind III and XbaI, with the Kozak sequence (GCCACC) added in front of the NaV coding sequence. The resulting constructs were named as pCMV-NaV1.5 and pCMV-NaV1.8. The rNaV1.4 and hNaV1.7 channels were cloned in the pCDNA3.1 plasmid, and the boundaries of the transmembrane domains were as presented in NCBI (NaV1.4^1^; NaV1.7^2^). We constructed the DCNaV1.8 channel by step-by-step substituting the transmembrane domains (D1 – 4) of NaV1.5 with those of NaV1.8. To take domain I (D1) substitution as an example, briefly, the pCMV-NaV1.5 plasmid was linearized by a pair of oppositely directed primers to delete the NaV1.5 D1, the NaV1.8 D1 was amplified by PCR by a pair of primer containing the upstream and downstream joint sequences. The linearized plasmid and the amplified domain segment were purified and ligated by using the recombinant cloning kit following the manufacturer’s instructions (CloneEZ^R^ PCR Cloning kit, Genscript, Nanjing, China). The primers for linearizing pCMV-NaV1.5 plasmid and for amplifying NaV1.8 transmembrane domains were as listed in our previous studies (Tang et al., 2015, 2017). We used the enzymatic digestion and ligation method to replace the loops L1, L2, L3, or L5 of NaV1.8 with that of NaV1.5, as well as the NaV1.8 L5 with that of NaV1.4 or NaV1.7. For construction of these chimeras, the sites Hind III, BglII and XbaI were engineered to flank these loops by site-directed mutations (deletion and insertion). In the chimera NaV1.5/1.8L5, the NaV1.5 C-terminus was substituted with that of NaV1.8 by using the engineered EcoR V site (between 5313 and 5314 bp in NaV1.5 mRNA numbering) and the Xbal I site. The introduced restriction sites in the coding sequence of NaVs were finally deleted by site-directed mutations. All constructs were sequenced to ensure that the correct chimeras were made.

### Plasmids Transfection

4 μg channel plasmid plus 0.5 μg pEGFP-N1 plasmid were co-transfected into HEK293T cells or ND7/23 cells by using lipofectamine 2000 following the manufacturer’s instructions (Invitrogen Corporation, Carlsbad, CA, United States). 4 – 6 h after transfection, cells were seeded onto PLL-coated coverslips. 24 h after transfection, cells were ready for patch-clamp analysis.

### Electrophysiology

Whole-cell currents of cells transfected with wild-type or chimeric NaV channels were recorded in an EPC-10 USB patch-clamp platform (HEKA Elektronik, Ludwigshafen, Germany). The recording pipets were pulled from glass capillaries (thickness = 0.225 mm) in a PC-10 puller (NARISHIGE, Tokyo, Japan). The pipet resistance was controlled to be 1.5 – 2.0 MΩ and only the tip was filled with pipet solution to minimize the fast capacitance. The standard pipet solution contains (in mM): 140 CsCl, 10 NaCl, 1 EGTA, 2 Mg-ATP, and 20 HEPES (pH = 7.4). Bath solution contains (in mM): 140 NaCl, 2 CaCl_2_, 1 MgCl_2,_ 5 KCl, 20 HEPES (pH = 7.4), and 10 Glucose. 300 nM TTX was added into the bath solution to block the endogenous NaVs when recording currents in ND7/23 cells. All chemicals were products of Sigma-Aldrich (Sigma-Aldrich, St. Louis, MO, United States) and dissolved in Milli-Q water. All experiments were conducted at room temperature (20 – 25°C). Data were collected by PatchMaster software and analyzed by Igo Pro 6.10A, OriginPro 8 and GraphPad Prism 5. The pipet capacitance and the cell capacitance were canceled by sequential fast and slow capacitance compensation by using the automatic computer-controlled circuit of the amplifier. To minimize the voltage error, the serial resistance (Rs) was controlled to be less than 10 MΩ and 80% Rs compensation was used, the speed value for Rs compensation was set to be 10 μs. The LockIn extension was used to measure the cell capacitance in case of measuring the current density. The dose-response curves were fitted by a Hill equation to estimate the potency (IC_50_) of the toxin. The NaVs repriming curves were fitted by a double exponential rising equation: y = a(1-e^-x/Tau1^) + c(1-e^-x/Tau2^), where Tau1 and Tau2 represented the fast and the slow time constant, respectively. The G-V and SSI curves were fitted by a Boltzmann equation: y = y_steady_ + (y_(0)_-y_steady_)/(1 + exp[(V-V_1/2_)/K]), where V_1/2_, V and K represented the midpoint voltage of kinetics, the test voltage and the slope factor, respectively.

### Data Analysis

Data were presented as MEAN ± SEM, n was presented as the number of separate experimental cells. Statistical significance was assessed by ONE-WAY ANOVA, multiple comparison between the groups was performed using Turkey method, statistical significance was accepted when *p* < 0.05.

## Results

### The Intracellular Loops Regulated NaV1.8 Functional Expression

**FIGURE 1:**
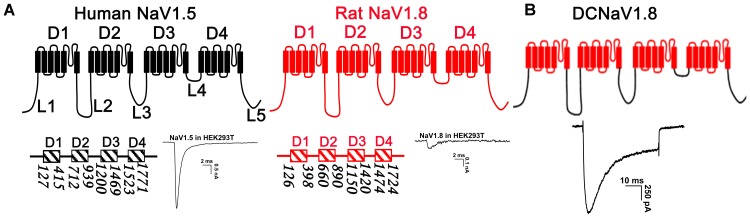
Strategy for NaV chimeras construction. **(A)** The topological structure of NaV1.5 and NaV1.8, the numbers indicated the border of the transmembrane domains, the representative current traces showed the NaV1.5 and NaV1.8 expressions in HEK293T cells. **(B)** Replacing the transmembrane domains of NaV1.5 with those of NaV1.8 constructed a chimeric channel (DCNaV1.8) functionally expressed in HEK293T cells. Currents were elicited by depolarizing to +10 mV from holding potential of –100 mV.

Voltage-gated sodium channels are tolerant of large module/modules substitution between different subtypes (Shaya et al., 2011; Tang et al., 2015). Our previous study showed that replacing D2, D3, or D4 of NaV1.5 with that of NaV1.8 constructed channels (NaV1.5/1.8D2, NaV1.5/1.8D3, and NaV1.5/1.8D4, respectively) functionally expressed in HEK293T cells (Tang et al., 2017). This inspired us to construct a chimeric channel which contains the intracellular loops of NaV1.5 and the transmembrane domains of NaV1.8, and it might express high-level functional currents in HEK293T cells. In **Figure 1A**, we determined the boundaries of the transmembrane domains (D1– 4) and the intracellular loops connecting them (L1 – 5), with the number bellow each domain indicating the first and the last amino acid, in NaV1.5 and NaV1.8 (NaV1.5^3^; NaV1.8^4^). The chimeric channel was constructed as described in the molecular cloning section and was named as DCNaV1.8. It expressed large NaV currents as the parental NaV1.5 channel when transfected into HEK293T cells (**Figure 1B**). And more than 70% (34/47) of the DCNaV1.8 transfected cells showed peak currents of 1 – 4 nA, the mean peak current density was -97.5 ± 10.0 pA/pF (*n* = 47). These data suggested that the NaV1.8 intracellular loops were responsible for its poor functional expression in HEK293T cells.

### C-terminus Substitution Made NaV1.8 Functional in HEK293T Cells

**FIGURE 2:**
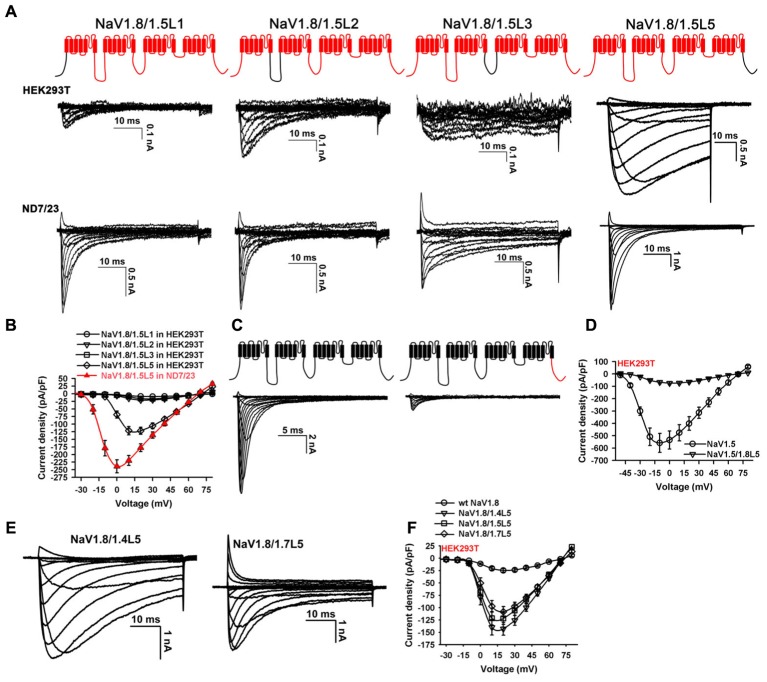
Screening analysis of NaV1.8 intracellular loops. **(A)** Chimeras were constructed by replacing each intracellular loop of NaV1.8 with that of NaV1.5, family currents were elicited by testing potentials from –80 mV to 80 mV, from holding of –100 mV. NaV1.8/1.5L5 but not others expressed large currents in HEK293T cells. **(B)**, Current densities of channels shown in **(A)**, the peak current density values for NaV1.8/1.5L5 expressed in HEK293T cells and ND7/23 cells were –121.9 ± 11.8 pA/pF and –239.0 ± 24.1 pA/pF, respectively, the difference was significant (*p* < 0.001, ONE-WAY ANOVA, *n* = 38 – 40). **(C)** NaV1.5/1.8L5 chimera (constructed as the cartoon illustrated), expressed much smaller currents than NaV1.5 in HEK293T cells. **(D)** Current densities of NaV1.5 and NaV1.5/1.8L5 in HEK293T cells, the peak values for NaV1.5 and NaV1.5/1.8L5 were –74.7 ± 11.7 pA/pF and –558.0 ± 76.9 pA/pF, respectively, the difference was significant (*p* < 0.001, ONE-WAY ANOVA, *n* = 33 – 34). **(E)** Representative traces showed replacing the C-terminus of NaV1.8 with that of NaV1.4 or NaV1.7 constructed channels (NaV1.8/1.4L5 and NaV1.8/1.7L5) functionally expressed in HEK293T cells. **(F)** Current densities of NaV1.8, NaV1.8/1.4L5, NaV1.8/1.5L5, and NaV1.8/1.7L5 in HEK293T cells, the peak values for NaV1.8/1.4L5 and NaV1.8/1.7L5 were –142.9 ± 12.7 pA/pF and –108.0 ± 10.4 pA/pF, respectively, the differences were significant between NaV1.8 and chimeric channels (*p* < 0.001, ONE-WAY ANOVA, *n* = 39 – 40). Unless otherwise indicated, data were presented as MEAN ± SEM.

Screening analysis was conducted to determine the key loop. Chimeras were made by substituting L1, L2, L3, or L5 of NaV1.8 with that of NaV1.5, respectively (NaV1.8/1.5L1, NaV1.8/1.5L2, NaV1.8/1.5L3, or NaV1.8/1.5L5; **Figure 2A**, upper panel). The NaV1.8 L4 was not included as it differs from the NaV1.5 L4 by only one amino acid residue. These channels were transfected into the HEK293T and ND7/23 cells, respectively, and family currents were elicited by series of 50-ms depolarizations from the holding potential of -100 mV (from -80 to +80 mV, in 10 mV increment). Representative traces showed that only the NaV1.8/1.5L5 channel expressed high-level functional currents in both cell lines. And the macroscopic currents showed that NaV1.8/1.5L5 inactivated much faster in ND7/23 cells than in HEK293T cells (**Figure 2A**). The poor expression of NaV1.8/1.5L1, NaV1.8/1.5L2, and NaV1.8/1.5L3 in HEK293T cells could not be explained by channel structural disorder caused by long sequence substitution, as they all could be functionally expressed in ND7/23 cells (**Figure 2A**). In HEK293T cells, the current density of NaV1.8/1.5L1, NaV1.8/1.5L2, or NaV1.8/1.5L3 at every testing voltage was less than -25 pA/pF (*n* = 38 – 40). However, the peak current density of NaV1.8/1.5L5 at +20 mV was -121.9 ± 11.8 pA/pF (**Figure 2B**; *p* < 0.001, when compared with NaV1.8/1.5L1, NaV1.8/1.5L2, and NaV1.8/1.5L3; ONE-WAY ANOVA, *n* = 38 – 40), and it showed left-shifted I-V relationship and higher current density in ND7/23 cells than in HEK293T cells (**Figure 2B**; peak current density value of -239.0 ± 24.1 pA/pF; *p* < 0.001, when compared with that in HEK293T cells; ONE-WAY ANOVA, *n* = 39). The screening analysis has confirmed that the NaV1.8 L5 (the C-terminus) was the key loop determining the functional expression of NaV1.8, and this conclusion was further supported by the reverse chimera NaV1.5/1.8L5 (constructed as the cartoon shown in **Figure 2C**). Its functional expression in HEK293T cells was compared with the parental NaV1.5 channel. The representative traces showed NaV1.5/1.8L5 expressed much smaller currents than NaV1.5 (**Figure 2C**). The peak current density values for NaV1.5 and NaV1.5/1.8L5 were -558.0 ± 76.9 pA/pF and -74.7 ± 11.7 pA/pF, respectively, the difference was significant (*p* < 0.001, ONE-WAY ANOVA, *n* = 33 – 34, **Figure 2D**).

The chimeric channels NaV1.8/1.4L5 and NaV1.8/1.7L5 were also made by substituting NaV1.8 L5 with that of NaV1.4 or NaV1.7, respectively. Their functional expressions in HEK293T cells were tested. The representative traces in **Figure 2E** showed that these two channels expressed large currents in HEK293T cells as NaV1.8/1.5L5. The peak current density values for NaV1.8/1.4L5 and NaV1.8/1.7L5 were -142.9 ± 12.7 pA/pF and -108.0 ± 10.4 pA/pF, respectively (**Figure 2F**, *n* = 40). The peak current density of NaV1.8/1.4L5, NaV1.8/1.5L5, or NaV1.8/1.7L5 at +20 mV was significantly higher than that of wild-type NaV1.8 (*p* < 0.001, ONE-WAY ANOVA, *n* = 39 – 40), while the differences between these three chimeric channels were not significant (*p* > 0.05, ONE-WAY ANOVA, *n* = 39 – 40). As the macroscopic currents of NaV1.8/1.7L5 mostly resembled that of wild-type NaV1.8 in ND7/23 cells, we focused on analyzing its electrophysiological and pharmacological properties.

### Gating Kinetics of NaV1.8/1.7L5 Channel

**FIGURE 3:**
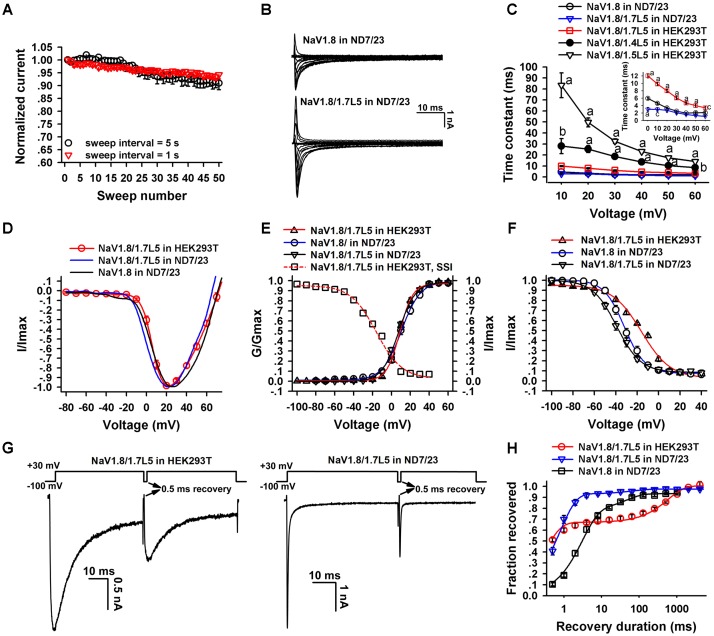
Electrophysiological properties of NaV1.8/1.7L5. **(A)** NaV1.8/1.7L5 currents in repetitive depolarizations. Currents elicited by 50 consecutive depolarizations to +10 mV were normalized to that of the first one, sweep interval was set to be 1 s (red triangel) or 5 s (black circle), *n* = 9 – 10. **(B)** Representative current traces of NaV1.8/1.7L5 and NaV1.8 expressed in ND7/23 cells. **(C)** The decay time constants of channels as shown, at the depolarizing voltages from 10 to 60 mV (a, b and c represented *p* < 0.001, *p* < 0.01, and *p* < 0.05, respectively; inset was an enlarged view of NaV1.8 and NaV1.8/1.7L5 traces; *n* = 15 in each group). **(D)**, I – V relationships for NaV1.8/1.7L5 expressed in HEK293T (red) or ND7/23 (blue) cells, and NaV1.8 expressed in ND7/23 cells (black), *n* = 9 – 14. **(E,F)** Steady-state activation and inactivation relationships of NaV1.8/1.7L5 expressed in HEK293T cells or ND7/23 cells, and NaV1.8 expressed in ND7/23 cells. The voltage dependence of steady-state inactivation was estimated by using a standard double-pulse protocol, in which a 50-ms depolarizing test potential to 20 mV followed a 500-ms prepulse (ranged from –100 mV to +40 mV, in 10 mV increment). *n* = 12 – 15. **(G)** Representative traces showed the repriming of NaV1.8/1.7L5 from fast inactivation after 0.5-ms holding at –100 mV, in HEK293T and ND7/23 cells. **(H)** Repriming kinetics of NaV1.8/1.7L5 expressed in either HEK293T(red) or ND7/23(blue) cells, and NaV1.8 expressed in ND7/23 cells (black), *n* = 7 – 14. Unless otherwise indicated, data were presented as MEAN ± SEM.

The gating kinetics of the NaV1.8/1.7L5 channel were analyzed and compared with that of the wild-type NaV1.8. We first tested the reproducibility of NaV1.8/1.7L5 currents during repetitive depolarizations. For a HEK293T cell transfected with NaV1.8/1.7L5, after the whole-cell configuration was established, series of 50-ms depolarizations to +20 mV from the holding potential of -100 mV were applied, the sweep intervals were 1 or 5 s, respectively. As shown in **Figure 3A**, the currents were very stable during repetitive depolarizations (*n* = 9 – 10). In ND7/23 cells, the currents of NaV1.8/1.7L5 resembled that of wild-type NaV1.8 (**Figure 3B**). Statistical analysis showed that, NaV1.8/1.7L5 inactivated much faster than wild-type NaV1.8 at 0 mV and +10 mV, but their inactivating rates from +20 mV to +60 mV were not significantly different. In HEK293T cells, NaV1.8/1.7L5 inactivated much slower than in ND7/23 cells at all voltages tested (**Figure 3C**, inset; a represented *p* < 0.001, c represented *p* < 0.05; ONE-WAY ANOVA, *n* = 15 in each group). And in HEK293T cells, NaV1.8/1.4L5 and NaV1.8/1.5L5 inactivated much slower than NaV1.8/1.7L5 (**Figure 3C**; a represented *p* < 0.001, b represented *p* < 0.01; ONE-WAY ANOVA, *n* = 15 in each group). The I-V relationships showed that the voltage-dependent activation of NaV1.8/1.7L5 channel in either ND7/23 or HEK293T cells was very similar to wild-type NaV1.8 channel in ND7/23 cells. The initial activation voltage and the peak current voltage were approximately -10 mV and +20 mV, respectively (**Figure 3D**, *n* = 9 – 14). As shown in **Figures 3E,F**, their G-V curves (steady-state activation curves) superimposed while their SSI curves (steady-state inactivation curves) showed differences, the fitting parameters of these curves were shown in **Table 1**. In HEK293T cells, the NaV1.8/1.7L5 activation and inactivation curves crossed at the voltage of 0 mV, at which approximately 20% of channels were activated, and the window current voltage ranged from -10 mV to +20 mV (**Figure 3E**, red solid and dashed lines). However, their repriming kinetics were distinct. As the voltage protocol shown in **Figure 3G**, there was a 0.5 ms holding to -100 mV between the pre-pulse and the test pulse. Few wild-type NaV1.8 channels could recover in the test pulse in such short -100 mV holding (current trace not shown). However, approximately 30–60% of the NaV1.8/1.7L5 currents recovered in the test pulse in both HEK293T and ND7/23 cells (**Figure 3G**). In **Figure 3H**, the proportion of the recovered currents was plotted as a function of the recovery duration and fitted with a two-exponential equation. In ND7/23 cells, the repriming of NaV1.8/1.7L5 was much faster than that of wild-type NaV1.8 within 64 ms (τ_fast_ was 0.6 ± 0.03 ms and 2.8 ± 0.3 ms; τ_slow_ was 37.6 ± 16.7 ms and 58.2 ± 12.0 ms, for NaV1.8/1.7L5 and wild-type NaV1.8, respectively; *p* < 0.001 and *p* > 0.05 for τ_fast_ and τ_slow_ comparison, respectively; ONE-WAY ANOVA, *n* = 7 – 11). The repriming of NaV1.8/1.7L5 in HEK293T cells was distinct from that in ND7/23 cells, it recovered much faster than wild-type NaV1.8 in 4 ms, but much slower between 4 to 1024 ms (τ_fast_ and τ_slow_ was 1.25 ± 0.24 ms and 674.8 ± 84.3 ms, respectively; *p* < 0.001, when compared with wild-type NaV1.8; ONE-WAY ANOVA, *n* = 14). These data suggested substituting the NaV1.8 C-terminus with that of NaV1.7 accelerated its recovery from inactivation, as well as the host cell lines could affect the kinetics.

**Table 1:** Fitting parameters of steady-state activation and inactivation curves of NaV1.8/1.7L5 and NaV1.8.

	Steady-state activation		Steady-state inactivation	
	V_a_ (mV)	Slope (mV)	V_*h*_ (mV)	Slope (mV)
NaV1.8/1.7L5 in HEK293T	9.1 ± 1.2	6.6 ± 0.3^∗∗∗^/***^###^***	–15.7 ± 1.2^∗∗∗^***^/###^***	13.4 ± 0.5^∗∗∗/###^
Nav1.8/1.7L5 in ND7/23	12.0 ± 2.2	9.3 ± 0.4	–40.0 ± 1.9	9.8 ± 0.8
Nav1.8 in ND7/23	7.7 ± 1.2	9.0 ± 0.3	–32.3 ± 1.7	8.5 ± 0.5

*^∗∗∗^p < 0.001, when compared with NaV1.8/1.7 L5 in ND7/23; ^###^p < 0.001, when compared with NaV1.8 in ND7/23; Data were presented as MEAN ± SEM, n = 12 –15.*

### NaV1.8/1.7L5 Resembled Wild-Type NaV1.8 in Blocking by MrVIB

**FIGURE 4:**
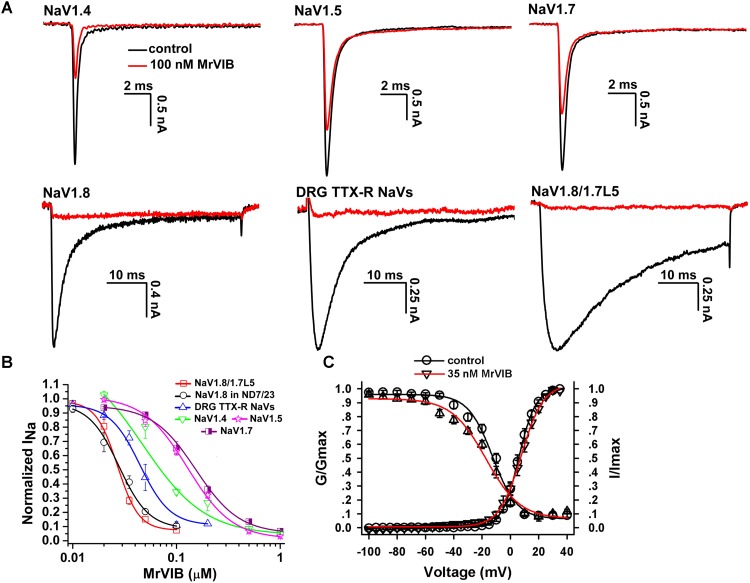
Effect of MrVIB on NaV1.8/1.7L5. **(A)** 100 nM MrVIB completely blocked the currents of DRG TTX-R NaVs, NaV1.8/1.7L5 expressed in HEK293T cells and NaV1.8 expressed in ND7/23 cells, and 100 nM MrVIB inhibited the currents of NaV1.4, NaV1.5, and NaV1.7 by 65.6 ± 2.1%, 37.4 ± 2.6%, and 34.8 ± 4.6%, respectively, *n* = 5 – 8. **(B)** Dose-response curves for MrVIB blocking channels as shown. Apparent IC_50_ values were determined as 26.9 ± 1.3 nM for NaV1.8/1.7L5, 27.9 ± 2.9 nM for NaV1.8, 49.3 ± 2.3 nM for DRG TTX-R NaVs, 65.1 ± 3.5 nM for NaV1.4, 127.8 ± 6.3 nM for NaV1.5 and 142.1 ± 10.0 nM for NaV1.7, respectively. *n* = 5 – 8. **(C)** MrVIB did not significantly alter the steady-state activation and inactivation kinetics of NaV1.8/1.7L5 (*n* = 6 – 8). Unless otherwise indicated, data were presented as MEAN ± SEM.

The pharmacological property of NaV1.8/1.7L5 was investigated and compared with that of wild-type NaV1.8, by testing their susceptibility to two NaV1.8 selective antagonists, MrVIB and APETx-2. As the representative traces shown in **Figure 4A**, 100 nM MrVIB inhibited NaV1.4, NaV1.5 and NaV1.7 currents by 65.6 ± 2.1%, 37.4 ± 2.6% and 34.8 ± 4.6%, respectively (*n* = 5 – 8). However, 100 nM MrVIB fully blocked the currents of wild-type NaV1.8 expressed in ND7/23 cell, NaV1.8/1.7L5 expressed in HEK293T cell, and the DRG TTX-R NaVs. These data were consistent with previous reports that MrVIB preferably targeted NaV1.8 channel. The dose-response curves of MrVIB blocking these channels showed that wild-type NaV1.8 and NaV1.8/1.7L5 were the same sensitive to MrVIB, while the curve for DRG TTX-R NaVs slightly shifted to right. The IC_50_ values were 27.9 ± 2.9 nM for NaV1.8, 49.3 ± 2.3 nM for DRG TTX-R NaVs and 26.9 ± 1.3 nM for NaV1.8/1.7L5, respectively (**Figure 4B**, *n* = 5 – 6). For NaV1.4, NaV1.5 and NaV1.7, the IC_50_ values were 65.1 ± 3.5 nM, 127.8 ± 6.3 nM and 142.1 ± 10.0 nM, respectively (*n* = 5 – 8). The G-V and SSI curves showed that MrVIB did not remarkably change the voltage-dependent activation and inactivation of NaV1.8/1.7L5 channel (**Figure 4C**, *n* = 6 – 8). These data suggested that the C-terminus substitution of NaV1.8 did not change its pharmacology.

### NaV1.8 Domain 2 and Domain 4 Were Involved in Binding With APETx-2

**FIGURE 5:**
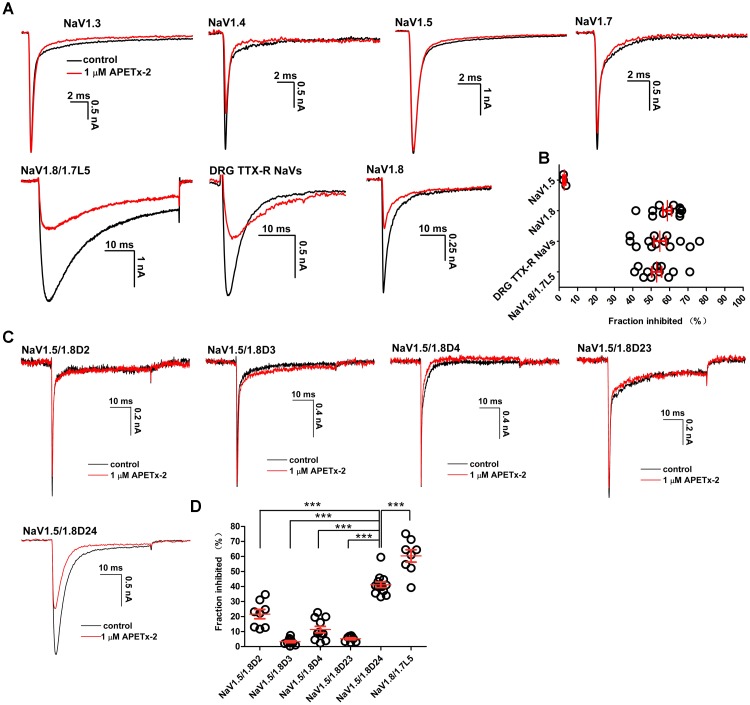
Mechanism of APETx-2 interacting with NaV1.8. **(A)** Representative traces showed the effects of APETx-2 on NaVs. NaV1.8/1.7L5, DRG TTX-R NaVs and NaV1.8 were much more susceptive to APETx-2 than NaV1.3 – 1.5 and NaV1.7. **(B)** Comparison of the inhibitory effect of 1 μM APETx-2 on channels as shown, a dot indicated the inhibition ratio from one tested cell, the ratio was 2.8 ± 0.5%, 58.8 ± 2.3%, 54.7 ± 3.3%, and 53.0 ± 2.7% for NaV1.5, NaV1.8, DRG TTX-R NaVs and NaV1.8/1.7L5, respectively. The differences were not significant between NaV1.8, DRG TTX-R NaVs and NaV1.8/1.7L5 (*p* > 0.05, ONE-WAY ANOVA, *n* = 11 – 13). **(C)** Representative traces showed the effect of APETx-2 on chimeras as shown, NaV1.5/1.8D24 was potently inhibited by 1 μM toxin. **(D)** 1 μM APETx-2 blocked NaV1.5/1.8D2, NaV1.5/1.8D3, NaV1.5/1.8D4, NaV1.5/1.8D23, NaV1.5/1.8D24 and NaV1.8/1.7L5 currents by 21.7 ± 3.1%, 3.3 ± 0.7%, 11.4 ± 2.1%, 5.2 ± 0.7%, 41.1 ± 1.9% and 60.0 ± 4.0%, respectively. The differences between NaV1.5/1.8D24 and other channels (NaV1.5/1.8D2, NaV1.5/1.8D3, NaV1.5/1.8D4, NaV1.5/1.8D23, and NaV1.8/1.7L5) were significant (*p* < 0.001, ONE-WAY ANOVA, *n* = 8 – 13). Unless otherwise indicated, data were presented as MEAN ± SEM.

Previous studies showed that the sea anemone toxin APETx-2 was an antagonist of AISC3 channel and NaV1.8 channel (Blanchard et al., 2012; Kozlov et al., 2012; Peigneur et al., 2012), which were two attractive targets for developing pain treating drugs (Wemmie et al., 2013; Bagal et al., 2014). As shown in **Figure 5A**, 1 μM APETx-2 did not affect NaV1.3 and NaV1.5 currents, and the NaV1.4 and NaV1.7 currents were partially inhibited but the ratio was less than 30%. However, 1 μM APETx-2 blocked the currents of NaV1.8 expressed in ND7/23 cell, NaV1.8/1.7L5 expressed in HEK293T cell and DRG TTX-R NaVs by approximately 50%. The inhibition ratios were 2.8 ± 0.5% for NaV1.5, 58.8 ± 2.3% for NaV1.8, 54.7 ± 3.3% for DRG TTX-R NaVs and 53.0 ± 2.7% for NaV1.8/1.7L5, respectively (**Figure 5B**, *n* = 11 – 13). The inhibition ratios for NaV1.8, DRG TTX-R NaVs and NaV1.8/1.7L5 were not statistically different (*p* > 0.05; ONE-WAY ANOVA, *n* = 11 – 13). We used the NaV1.5/NaV1.8 chimeric channels to study the molecular mechanism of NaV1.8-APETx-2 interaction. The single-domain chimeras (NaV1.5/1.8D2, NaV1.5/1.8D3, and NaV1.5/1.8D4) were as previously reported (Tang et al., 2017), and two double-domains chimeras (NaV1.5/1.8D23 and NaV1.5/1.8D24) were constructed in this study. 1 μM APETx-2 showed different activity on these channels, with the NaV1.5/1.8D2, NaV1.5/1.8D3, NaV1.5/1.8D4, and NaV1.5/1.8D23 currents being slightly or moderately inhibited, while the NaV1.5/1.8D24 current being robustly inhibited (**Figure 5C**). As shown in **Figure 5D**, the inhibition ratios were 21.7 ± 3.1% for NaV1.5/1.8D2 (*n* = 8), 3.3 ± 0.7% for NaV1.5/1.8D3 (*n* = 10), 11.4 ± 2.1% for NaV1.5/1.8D4 (*n* = 11), 5.2 ± 0.7% for NaV1.5/1.8D23 (*n* = 8), 41.1 ± 1.9% for NaV1.5/1.8D24 (*n* = 13) and 60.0 ± 4.0 for NaV1.8/1.7L5 (*n* = 8), respectively. The differences were significant between NaV1.5/1.8D24 and the single-domain chimeras, as well as between NaV1.5/1.8D24 and NaV1.8/1.7L5 (*p* < 0.001, ONE-WAY ANOVA). These data suggested that at least the NaV1.8 domain 2 and domain 4 were involved in binding with APTEx-2.

## Discussion

The ion channels and receptors represented two primary drug targets in the cell membrane, and their high-level functional expression in mammalian cell lines was of particular importance for their pharmacological and biophysical researches. Lots of methods were used to overexpress ion channels and receptors. As in the case of M_3_ muscarinic acetylcholine receptor (M3R), its functional expression in mammalian cells were substantially increased by codon optimization, fusing tags with the protein, and using virus as the transfection vector (Romero-Fernandez et al., 2011). Our previous study also showed that the NaV1.9 functional expression in ND7/23 cells could be enhanced by fusing an EGFP tag to its C-terminus (Zhou et al., 2017). Another strategy was to use pharmacological chaperones, as shown in previous reports that atropine and lidocaine increased the functional expression of M3R and NaV1.8, respectively (Ward et al., 2002; Zhao et al., 2007). The right choice of the host cell line was also important. As for NaV1.8, it expressed functional currents in ND7/23 and SH-SY5Y cells but not in non-neuronal cell lines. However, the ND7/23 cells expressed abundant endogenous NaVs (Rogers et al., 2016), TTX must be used in the bath solution to separate the recombinant NaV1.8 currents. Finally, Co-expressing the accessory proteins and even the expression temperature could affect the expression level of ion channels, as shown in the case of NaV1.9 channel expressed in the HEK293T cells (Lin et al., 2016). Future works could attempt to express NaV1.8 and NaV1.9 channels by virus transfection or codon optimization.

The *in vivo* expressions of ion channels in neurons were subtly tuned and the dysregulations were closely related to diseases, as those of NaVs in neuropathic pain and ASIC1a in ischemia (Lai et al., 2003; Chai et al., 2010). It was shown that some motifs in ion channels were important in determining their subcellular locations (Standley et al., 2000; Xia et al., 2001; Garrido et al., 2003). In this study, by conducting a screening analysis, we confirmed that the NaV1.8 C-terminus was the limiting factor for its poor functional expression in HEK293T cells. Interestingly, a recent study showed that substituting the NaV1.9 C-terminus with that of NaV1.4 also constructed chimeric channel functionally expressed in various host cells (Goral et al., 2015). It remains to be elucidated if the NaV1.8 C-terminus functioned by ER retention, or on the other hand, by fast internalization mechanism. The second means that the NaV1.8 proteins were fast retrieved from membrane by internalization signals, as that of NaV1.2 channel in dendrites in hippocampal neurons (Garrido et al., 2001). It is of particular interest to investigate the *in vivo* role of NaV1.8 C-terminus in regulating channel expression.

The NaV1.8/1.7L5 channel, constructed by replacing NaV1.8 C-terminus with that of NaV1.7, expressed high-level functional currents in HEK293T cells by transient transfection, and this channel pharmacologically resembled wild-type NaV1.8. The screening analysis of the NaV1.8 intracellular loops provided us with more solid evidence that the role of the NaV1.8 C-terminus is unique. This expression system could be used in the electrophysiological and pharmacological studies of NaV1.8 channel, especially in analyzing the gating kinetics of disease-associated NaV1.8 mutants and in investigating the molecular mechanism of drug-NaV1.8 interaction by mutating channel. The peak current density of NaV1.8/1.7L5 in this study was about 2-folds of that reported by Vijayaragavan et al. (2004), possibly because we used the human NaV1.7 rather than the rat NaV1.7 as the parental channel, as sequence alignment revealed approximately 9% variation between their C-termini. In addition, sequence alignment showed the C-termini of human Nav1.7 and rat NaV1.8 had an identity of 54%, while that for rat NaV1.7 and rat NaV1.8 is 51.9%. When compared with those of NaV1.8/1.4L5 and NaV1.8/1.5L5 channels (**Figures 2A,E**), the macroscopic currents of NaV1.8/1.7L5 channel showed apparently accelerated inactivation. This is not surprising as the NaV C-terminus was proposed to interact with the inactivation particle (the D3 – 4 linker) by using the calcium sensor calmodulin as a bridge (Sarhan et al., 2012) or its conformation change might directly affect the D4 transmembrane domain, which is a key determinant of NaV fast inactivation (Wang et al., 2014). Lots of electrophysiological studies have shown that swapping the C-terminus between NaV subtypes or mutation in the C-terminus region changed channels’ inactivation kinetics (Deschenes et al., 2001; Mantegazza et al., 2001; Kass, 2006; Lee and Goldin, 2008; Nguyen and Goldin, 2010). We speculated that the sequence variation between different NaV C-termini is responsible for the observed differences in the inactivation kinetics of these three chimeric channels. However, the exact mechanism is currently unknown and requires further research.

We used MrVIB and APETx-2 to investigate the pharmacology of NaV1.8/1.7L5 and compared it with that of wild-type NaV1.8. Although we did not test more toxins or chemicals, we speculated that drugs targeting the extracellular/transmembrane parts of NaV1.8 should act on the NaV1.8/1.7L5 channel in the same way, as the structure of NaV is largely stabilized by its transmembrane domains. The dose-response curve for MrVIB blocking DRG TTX-R NaVs was slightly shifted when compared with those of NaV1.8 and NaV1.8/1.7L5. It might be explained by endogenous and heterologous expressed channels showing different susceptibility to the toxin. In fact, previous study showed that the MrIVB activity on NaV1.8 could be modulated by beta subunits (Wilson et al., 2011). In addition, by using the panel of NaV1.5/NaV1.8 chimeras, we showed that APETx-2 is not a classical NaV site 4 toxin (**Figure 5**), and the detail of the toxin binding site in NaV1.8 is still to be investigated.

## Author Contributions

CT and ZL designed the experiments and wrote the manuscript. CT, YZ, DT, and XZ performed the experiments and the data analysis. SL and PC helped to perform the data analysis.

## Conflict of Interest Statement

The authors declare that the research was conducted in the absence of any commercial or financial relationships that could be construed as a potential conflict of interest. The reviewer JE and handling Editor declared their shared affiliation.

## Footnotes

1 https://www.ncbi.nlm.nih.gov/protein/NP_037310.1

2 https://www.ncbi.nlm.nih.gov/protein/NP_002968.1

3 https://www.ncbi.nlm.nih.gov/protein/NP_000326.2

4 https://www.ncbi.nlm.nih.gov/protein/NP_058943.1

## ABBREVIATIONS

NaV: voltage-gated sodium channel
PM: pore module
VSM: voltage sensing module.
